# Evaluating the impact of stay-at-home and quarantine measures on COVID-19 spread

**DOI:** 10.1186/s12879-022-07636-4

**Published:** 2022-07-27

**Authors:** Renquan Zhang, Yu Wang, Zheng Lv, Sen Pei

**Affiliations:** 1 School of Mathematical Sciences, Dalian University of Technology, 116024 Dalian, China; 2 School of Control Science and Engineering, Dalian University of Technology, 116024 Dalian, China; 3 Department of Environmental Health Sciences, Mailman School of Public Health, Columbia University, 10032 New York, USA

**Keywords:** COVID-19, Compartmental model, Non-pharmaceutical interventions, Data assimilation

## Abstract

**Background:**

During the early stage of the COVID-19 pandemic, many countries implemented non-pharmaceutical interventions (NPIs) to control the transmission of SARS-CoV-2, the causative pathogen of COVID-19. Among those NPIs, stay-at-home and quarantine measures were widely adopted and enforced. Understanding the effectiveness of stay-at-home and quarantine measures can inform decision-making and control planning during the ongoing COVID-19 pandemic and for future disease outbreaks.

**Methods:**

In this study, we use mathematical models to evaluate the impact of stay-at-home and quarantine measures on COVID-19 spread in four cities that experienced large-scale outbreaks in the spring of 2020: Wuhan, New York, Milan, and London. We develop a susceptible-exposed-infected-removed (SEIR)-type model with components of self-isolation and quarantine and couple this disease transmission model with a data assimilation method. By calibrating the model to case data, we estimate key epidemiological parameters before lockdown in each city. We further examine the impact of stay-at-home and quarantine rates on COVID-19 spread after lockdown using counterfactual model simulations.

**Results:**

Results indicate that self-isolation of susceptible population is necessary to contain the outbreak. At a given rate, self-isolation of susceptible population induced by stay-at-home orders is more effective than quarantine of SARS-CoV-2 contacts in reducing effective reproductive numbers $R_e$. Variation in self-isolation and quarantine rates can also considerably affect the duration of outbreaks, attack rates and peak timing. We generate counterfactual simulations to estimate effectiveness of stay-at-home and quarantine measures. Without these two measures, the cumulative confirmed cases could be much higher than reported numbers within 40 days after lockdown in Wuhan, New York, Milan, and London.

**Conclusions:**

Our findings underscore the essential role of stay-at-home orders and quarantine of SARS-CoV-2 contacts during the early phase of the pandemic.

**Supplementary Information:**

The online version contains supplementary material available at 10.1186/s12879-022-07636-4.

## Background

Emerged in late 2019, a new respiratory pathogen, SARS-CoV-2, rapidly spread across the globe and caused a global pandemic. As of June 1, 2022, more than 527 million confirmed cases have been reported worldwide, of which more than 6.2 million have died [1]. The disease caused by SARS-CoV-2, the coronavirus disease 2019 (COVID-19), is characterized by a substantial proportion of infections with mild or no symptoms [2] and a strong age gradient in the risk of death [3, 4]. During the early stage of the COVID-19 outbreak, the number of confirmed cases generally followed an exponential increase. Studies have shown that the average estimate of the basic reproductive number $R_0$ is between 2.24 and 3.58 [5]. After China implemented strict control measures, the spread of COVID-19 within China was greatly reduced [6–10]. In other countries, after the initial reporting of infection cases, NPIs such as suspension of classes, cessation of large-scale gatherings, and closure of entertainment and leisure venues have been adopted. These control measures were estimated to effectively slow down the community transmission of SARS-CoV-2 [11–13].

Before the development of vaccine and its wide administration, NPIs are the primary means to reduce the spread of SARS-CoV-2 [14–16]. During vaccination campaign, NPIs also remain key in reducing infections [17, 18]. Among the implemented NPIs, stay-at-home orders were announced to encourage self-isolation of all population to reduce potential contacts with infectious individuals. In parallel, quarantine was used to separate individuals who have been exposed to COVID-19 from others, which prevents spread of COVID-19 that can occur before a person knows they are infected. During the early days of the COVID-19 pandemic, case isolation and contact tracing were employed to contain the outbreak; however, for an infectious disease whose infectiousness begins before symptoms appear, the effectiveness of isolating cases and tracing contacts is limited [19–21]. Indeed, a study found that, for the Lombardy ICU network in Italy, strict self-quarantine measures may be the only possible way to contain the spread of infection [22]. As a result, understanding the impact of self-isolation induced by stay-at-home orders and quarantine of SARS-CoV-2 contacts on COVID-19 spread and the intensity of these measures required to contain an outbreak is critical for planning control measures by governments and public health authorities.

In this study, we developed a mathematical model to estimate the effect of stay-at-home and quarantine on suppressing COVID-19 spread in four cities: Wuhan in China, New York City in the US, Milan in Italy, and London in the UK. Those cities experienced early outbreaks of COVID-19 and all enforced strict interventions to control the transmission of SARS-CoV-2. We incorporated components of self-isolation and quarantine into a classical susceptible-exposed-infected-removed (SEIR) model, and calibrated the model to confirmed cases in each city during the early phase of the pandemic using a data assimilation method. We estimated time-varying key epidemiological parameters before lockdown in each city, and evaluated the impact of the isolation rates of susceptible, exposed and undetected infected populations on disease transmission. Particularly, we estimated the required minimal self-isolation and quarantine rates of those populations to reduce the effective reproductive number below 1 at the beginning of lockdown. We further simulated counterfactual outbreaks within 40 days following lockdown, assuming no stay-at-home and quarantine were implemented in those cities, and estimated the averted cases attributed to these two measures. Overall, stay-at-home and quarantine measures have effectively prevented 3,589,622, 3,281,480, 629,046 and 2,452,750 reported cases during this 40-day period in Wuhan, New York, Milan, and London, respectively. In other words, without these two measures, the cumulative cases during the 40-day period could be 71, 21, 41, and 99 times higher than the reported cases in these four cities.

## Methods

### Model

**Fig. 1:**
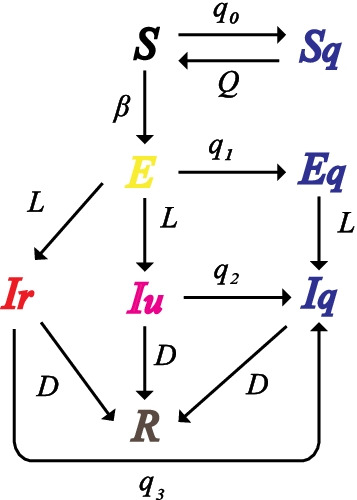
Dynamics of the transmission model. The compartments *S*, *E*, $I_r$, $I_u$ and *R* represent susceptible, exposed, reported infected, unreported infected and removed populations. $S_q$, $E_q$ and $I_q$ are susceptible, exposed and infected individuals under quarantine. $q_0$ is the self-isolation rate of susceptible persons; $q_1$ is the quarantine rate of exposed persons; $q_2$ is the quarantine rate of unreported infections; and $q_3$ is the isolation rate of confirmed cases. $\beta$ is the transmission rate of SARS-CoV-2, *L* is the mean duration of latency period, *D* is the mean duration of infectious period, and *Q* is the average length of self-isolation

We used a modified SEIR model to depict the transmission of SARS-CoV-2 in a location with quarantine measures. The model dynamics is shown in Fig. 1. Specifically, *S*, *E*, $S_q$, $E_q$, $I_r$, $I_u$, $I_q$ and *R* represent susceptible, exposed, self-isolated susceptible, quarantined exposed, reported infected, unreported infected, isolated infected and removed (recovered or dead) populations. A susceptible individual can be infected by a reported infection with a transmission rate $\beta$ or an unreported infection with a transmission rate $\mu \beta$ where $\mu \in [0,1]$. Note we assume undocumented infections are less contagious than confirmed cases, as indicated in previous studies [23–26]. Exposed individuals become contagious after a mean latency period of *L* days. A fraction of infected population, $\alpha$, is ascertained as confirmed cases. Infected individuals recover or die after a mean infectious period of *D* days.

We assume susceptible population is self-isolated by a rate $q_0$, representing the effect of stay-at-home orders. Quarantine of SARS-CoV-2 contacts has little impact on the overall self-isolation rate of susceptible population during the early pandemic as the percentage of exposed contacts is negligible compared to the total population. For instance, in a city with millions of residents, a few thousand exposed contacts only account for less than 0.1% of total population. We therefore neglect the effect of quarantine of SARS-CoV-2 contacts on susceptible population. We assume the self-isolation rate $q_0$ for susceptible population does not depend on infectious population as the proportion of total population who have contacts with infections is negligible. This assumption simplifies the model structure and does not significantly affect the results. For exposed individual, we assume the quarantine rate is $q_1$, representing the effect of quarantine. Undocumented infections are isolated following a different isolation rate $q_2$ to reflect the differential perception of infection risk as they may have mild symptoms. In total, we define three separate rates $q_0$, $q_1$ and $q_2$ for susceptible, exposed, and undocumented infections respectively. Confirmed cases are isolated following an isolation rate $q_3$ after they become infectious. Self-isolated susceptible people are released from self-isolation after an average of *Q* days. We assume self-isolated or quarantined individuals (susceptible, exposed or infected) do not participate in disease transmission. The transmission dynamics is described by the following equations:

1 $$\begin{aligned}&\frac{dS}{dt}=-\left[ q_0S+(1-q_0)\left( \frac{\beta I_rS}{N}+\frac{\mu \beta I_uS}{N}\right) \right] +\frac{S_q}{Q} \end{aligned}$$

2 $$\begin{aligned}&\frac{dE}{dt}=(1-q_0)\left( \frac{\beta I_rS}{N}+\frac{\mu \beta I_uS}{N}\right) -q_1E-(1-q_1)\frac{E}{L} \end{aligned}$$

3 $$\begin{aligned}&\frac{dE_q}{dt}=q_1E-\frac{E_q}{L} \end{aligned}$$

4 $$\begin{aligned}&\frac{dS_q}{dt}=q_0S-\frac{S_q}{Q} \end{aligned}$$

5 $$\begin{aligned}&\frac{dI_r}{dt}=\alpha (1-q_1)\frac{E}{L}-\frac{I_r}{D}-q_3I_r \end{aligned}$$

6 $$\begin{aligned}&\frac{dI_u}{dt}=(1-\alpha )(1-q_1)\frac{E}{L}-\frac{I_u}{D}-q_2I_u \end{aligned}$$

7 $$\begin{aligned}&\frac{dI_q}{dt}=\frac{E_q}{L}-\frac{I_q}{D}+q_2I_u+q_3I_r \end{aligned}$$

8 $$\begin{aligned}&\frac{dR}{dt}=\frac{I_r}{D}+\frac{I_u}{D}+\frac{I_q}{D} \end{aligned}$$

Using model equations, we compute the effective reproductive number, $R_e$, i.e. the average number of new infections caused by a single infected individual in a population with partial immunity, as

9 $$\begin{aligned} R_e=\left( \frac{(1-q_1)\alpha \beta }{q_3+\frac{1}{D}}+\frac{(1-q_1)(1-\alpha ) \mu \beta }{q_2+\frac{1}{D}}\right) \frac{S}{N}. \end{aligned}$$

In model simulations, we deterministically integrate equations using the 4th-order Runge-Kutta method.

### Model calibration before lockdown

We calibrated the transmission model to daily confirmed cases in each city using a data assimilation method - the ensemble adjustment Kalman filter (EAKF) [27]. We chose to use case data because they are available for most locations as a standard surveillance target. To account for case underreporting, we explicitly modeled undocumented infections in our model, which can partially alleviate the effect of limited testing resources. Hospitalization data are less impacted by underreporting; however, hospitalization data have a longer delay compared with case data and are more biased to older and vulnerable population. To match infection to hospitalization in the transmission model, an additional infection-hospitalization rate (IHR) needs to be defined. This IHR may vary in different locations due to different levels of healthcare capacity and could further complicate the model.

The EAKF is a recursive filtering technique that assimilates observations into a dynamic model to generate a posterior estimate of model state (both parameters and variables). Importantly, the EAKF can estimate time-varying parameters, as model parameters are updated daily once new information of confirmed cases is available. This capability is critical for this study because model parameters such as the transmission rate and ascertainment rate may change over time due to varying control measures and testing availability. In this study, we estimated the posterior distributions of model parameters for each day, reflecting the shifting situation during the early pandemic. The EAKF has been widely used in numerical weather prediction [27, 28] as well as inference and forecasting of infectious diseases such as influenza [29–33], COVID-19 [34–38], other respiratory viruses [39], and antimicrobial-resistant pathogens [40, 41].

The EAKF assumes a Gaussian distribution of both the prior and likelihood and adjusts the prior distribution to a posterior using Bayes’ rule. To represent the state-space distribution, the EAKF maintains an ensemble of system state vectors acting as samples from the distribution. In particular, the EAKF assumes that both the prior distribution and likelihood are Gaussian, and thus can be fully characterized by their first two moments (mean and variance). The update scheme for ensemble members is computed using Bayes’ rule (posterior $\propto$ prior $\times$ likelihood) via the convolution of the two Gaussian distributions. In the EAKF, variables and parameters are updated deterministically such that the higher moments of the prior distribution are preserved in the posterior. Details on the implementation of the EAKF can be found in published studies [27, 42].

In the analysis, we first focus on the period before lockdown and stay-at-home order were announced in each city. For Wuhan, New York, Milan and London, we used daily case data reported from January 16 to January 23, March 1 to March 19, February 25 to March 8, and March 6 to March 23, respectively. We collected the daily reported case data in the four cities (see details in Availability of data and materials). To mitigate the impact of possible irregular reporting during the early stage of the pandemic, we used a five-day moving average to smooth the epidemic curves. Before lockdown, confirmed cases were isolated but population-level stay-at-home orders were not yet affected. We therefore set the self-isolation rate $q_0$ as zero. To reduce the number of unknown parameters, we fixed several parameters estimated in previous studies. Specifically, the isolation rate of confirmed cases $q_3$ is estimated as the reciprocal of the delay from the onset of contagiousness to confirmation. For instance, the confirmation delay in Wuhan was estimated to be 6-10 days [2, 43]; we therefore set the range of $q_3$ to be [0.1, 0.16] for Wuhan. Confirmation delays in New York, Milan and London were also reported in previous studies [21, 43–45]. We used these estimates to define $q_3$ in these cities. Certain exposed individuals and unreported infections may be quarantined before the lockdown. We fixed the range of quarantine rates $q_1$ and $q_2$ as [0.05, 0.1] in the model. We further fixed relative transmission rate $\mu$, the mean latency period *L* and mean infectious period *D* as reported in other studies [2]. In model simulations and inference before lockdown, these parameters were uniformly drawn from the prior range and were fixed throughout the analysis. We used the model-inference framework to estimate two parameters: $\alpha$, $\beta$. The fixed ranges of $q_1$, $q_2$, $q_3$, $\mu$, *L* and *D* and the prior ranges of $\alpha$, $\beta$ were shown in Table 1. We used 300 ensemble members in the EAKF, and drew initial parameters uniformly from the prior ranges.

### Counterfactual simulations after lockdown

We estimated the number of COVID-19 cases averted by stay-at-home and quarantine measures after lockdown using counterfactual simulations. We first employed the model-inference system to estimate daily posterior model parameters within 40 days after lockdown in each city. Specifically, we estimated the daily transmission rate $\beta$, ascertainment rate $\alpha$, self-isolation rate $q_0$, quarantine rates $q_1$ and $q_2$, isolation rate of confirmed cases $q_3$, relative transmissibility of undocumented infections $\mu$, mean latency period *L*, and mean infectious duration *D*. We further fixed the average length of self-isolation *Q* as 75 (from January 24 to April 7), 80 (from March 20 to June 7), 56 (from March 9 to May 3) and 48 (from March 24 to May 10) days for Wuhan, New York, Milan and London, respectively. The parameter inference was performed for 100 realizations independently, each with different initialization of the ensemble members in the EAKF, to obtain parameter combinations that fit the observed case data. Note, we estimated the time-varying parameters after lockdown to reflect changing control measures and testing practice.

We then plugged in the estimated daily parameters and ran model simulations for 40 days, for which we varied the self-isolation rate $q_0$ and quarantine rates $q_1$ and $q_2$. In counterfactual simulations, we tested the following two scenarios. In the first, we set $q_0=q_1=q_2=0$ after lockdown, assuming no stay-at-home orders and quarantine were implemented. In this analysis, we focus on the combined effect of stay-at-home orders and quarantine. In the second, we only set $q_0=0$ and keep $q_1$ and $q_2$ unchanged as the estimated values. This counterfactual simulation estimates the effect of stay-at-home orders. The counterfactual simulations can inform the number of COVID-19 cases averted by stay-at-home orders and quarantine measures. Note in counterfactual simulations, we only lifted stay-at-home and quarantine for susceptible, exposed, and unreported infections. Other model parameters, such as the time-varying transmission rate and ascertainment rate, remain the same as estimated in the model.

## Results

### Epidemiological characteristics before lockdown

**Fig. 2:**
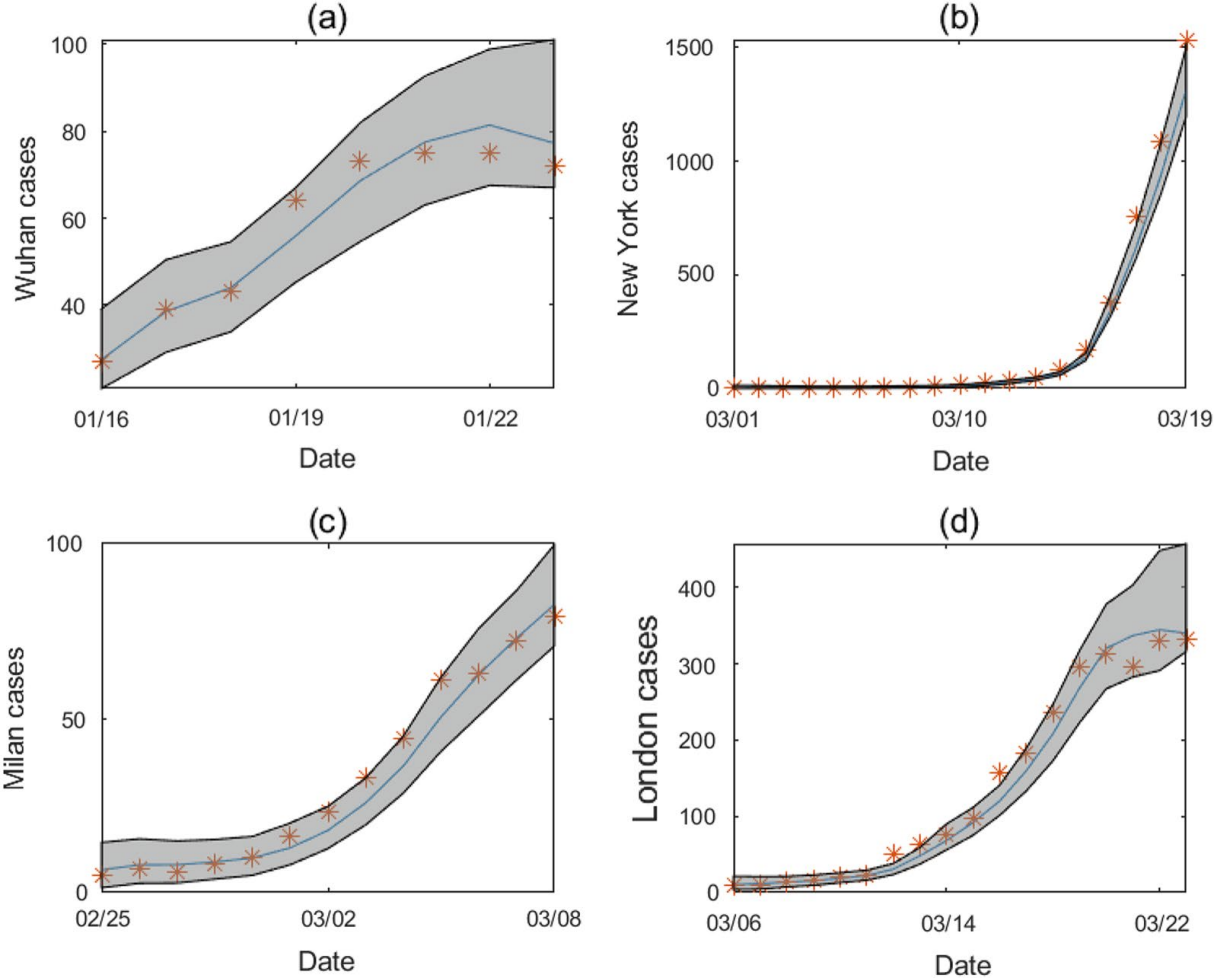
Model fitting for **a** Wuhan from January 16 to January 23, (**b**) New York from March 1 to March 19, **c** Milan from February 25 to March 8, and **d** London from March 6 to March 23. The orange star symbol represents reported case number, the blue curve is the mean posterior fitting using the EAKF, and the gray region shows the 95% CI

Using the model-data assimilation framework, we estimated the posterior epidemiological parameters in the four cities before lockdown (see Table 2). The posterior fitting agrees well with observed daily cases, as shown in Figure 2. Before lockdown, the number of new cases increased rapidly during the study period, and most data points fall within the 95% CI. The estimated effective reproductive number $R_e$ is generally in line with previous estimates [5]. New York has the highest estimated $R_e=2.89$, followed by London ($R_e=2.80$), Milan ($R_e=2.70$) and Wuhan ($R_e=2.25$). Only $8.6\%$ infections were estimated to be confirmed in New York, agreeing with previous modeling results [35] and surveys of healthcare-seeking behavior [46] and seroprevalence [47]. For Wuhan, a seroprevalence study found that the ascertainment rate before April 2020 was about 6.8% [48], which is close to our estimate of 7.4%. We also estimated the ascertainment rates in Milan and London to be 7.4% and 7.6%, respectively. Serological surveys in Milan [49] and London [50] resulted in 7.1% and 7.1% ascertainment rates, generally matching our estimates.

**Table 1:** Prior parameter range for Wuhan, New York, Milan, and London before lockdown

	Wuhan	New York	Milan	London	
$q_1$	(0.05,0.1)	(0.05,0.1)	(0.05,0.1)	(0.05,0.1)	Fixed
$q_2$	(0.05,0.1)	(0.05,0.1)	(0.05,0.1)	(0.05,0.1)	Fixed
$q_3$	(0.1,0.16)	(0.07,0.11)	(0.12,0.5)	(0.1,0.16)	Fixed
$\beta$	(1,2)	(1,2)	(1,2)	(1,2)	Estimated
$\mu$	(0.45,0.65)	(0.45,0.65)	(0.45,0.65)	(0.45,0.65)	Fixed
$\alpha$	(0.02,0.12)	(0.02,0.12)	(0.02,0.12)	(0.02,0.12)	Estimated
*L*	(3,5)	(3,5)	(3,5)	(3,5)	Fixed
*D*	(3,5)	(3,5)	(3,5)	(3,5)	Fixed

### Impact of stay-at-home and quarantine rates on COVID-19 spread

We use model simulations to examine the minimal self-isolation rate $q_0$ and quarantine rates $q_1$ and $q_2$ required to reduce $R_e$ below one. Estimates of these threshold values are important from a public health point of view. First, these two rates can be changed by the compliance with policies. If necessary, local governments can enforce stricter policies and adopt more effective contact tracing to increase the stay-at-home and quarantine rates to reduce $R_e$ below one. Second, in settings where these two rates cannot be modified, the threshold can indicate whether it is possible to contain the outbreak through stay-at-home and quarantine alone. The threshold values can be used to assess the controllability of the disease.

**Table 2:** Posterior parameter estimates of Wuhan, New York, Milan, and London before lockdown

	Wuhan	New York	Milan	London
$\beta$	1.40	1.78	1.74	1.74
$\alpha$	0.074	0.086	0.074	0.076
$R_e$	2.25	2.89	2.70	2.80
95% CI	(1.61,3.07)	(2.13,3.69)	(1.91,3.56)	(2.14,3.54)

We initialized the transmission model using model states and parameters inferred on the last day before lockdown (as shown in Table 2), varied self-isolation rate $q_0$ (caused by stay-at-home orders) and the quarantine rates $q_1$ (the quarantine rate of exposed individuals) and $q_2$ (the quarantine rate of unreported infections), and ran model simulations until the outbreak stops. We examined the effects of different combinations of self-isolation and quarantine rates on the effective reproductive number, the duration of outbreak, the attack rate, and peak timing. For the ease of visualization in 2D plots, we fixed one parameter and varied the other two in model simulations. The choice of the fixed parameter values is arbitrary and does not impact the qualitative results.

**Fig. 3:**
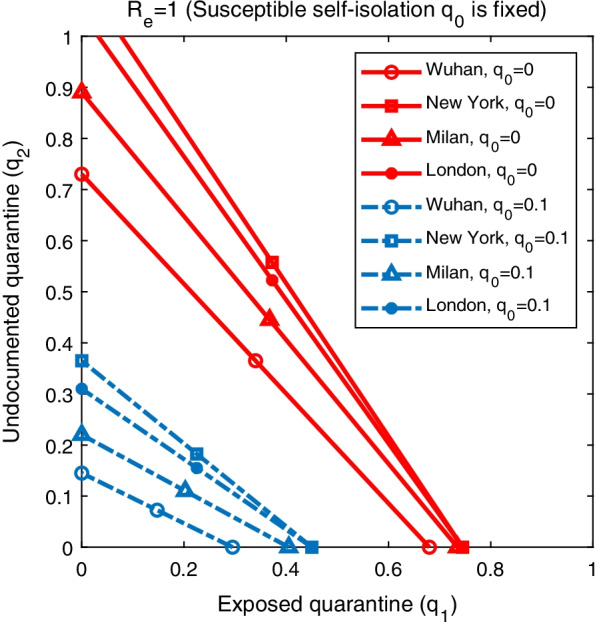
The combination of $q_1$ and $q_2$ that lead to $R_e=1$ in Wuhan, New York, Milan, and London. The solid lines represent results obtained for $q_0=0$, while the dash lines are the results for $q_0=0.1$. Other parameters are set as in Tables 1, 2

Figure 3 shows the impact of quarantine rates $q_1$ and $q_2$ on the effective reproductive number $R_e$ on the first day of model simulation for fixed self-isolation rates $q_0=0$ (solid lines) and $q_0=0.1$ (dash lines). The figure shows the combinations of $q_1$ and $q_2$ that lead to $R_e=1$ in the four cities. The results were obtained for $q_3$ set as in Table 1, i.e. the same isolation rate of confirmed cases as before lockdown. If susceptible individuals are not self-isolated (i.e., no stay-at-home orders) and control measures on confirmed cases remain the same after lockdown, quarantining only unreported infections is not sufficient to contain the outbreak in New York and London - even with $q_2=1$, the effective reproductive number $R_e$ is still above one. For Wuhan and Milan, it is possible to reduce $R_e$ below one through the quarantine of only undetected infections, but the majority of undocumented cases need to be quarantined quickly ($q_2$ is close to 1). In reality, this is very challenging because rapid testing is not widely available and the turnaround time of PCR testing is too long to support timely quarantine. As a result, it is necessary to self-isolate susceptible population in order to control the outbreak. Model simulations also indicate that, for $q_0=0.1$, self-isolation of susceptible population can substantially reduce the required quarantine rates $q_1$ and $q_2$ for $R_e<1$, suggesting that stay-at-home orders are more effective in reducing effective reproductive numbers.

**Fig. 4:**
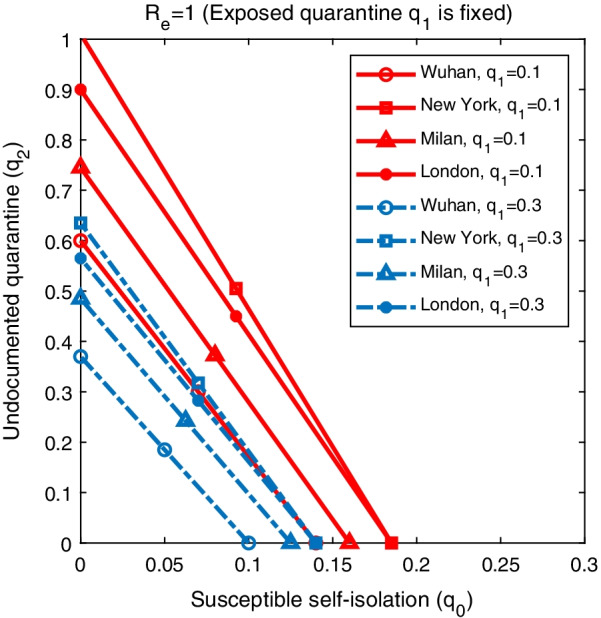
The combination of $q_0$ and $q_2$ that lead to $R_e=1$ in Wuhan, New York, Milan, and London. The solid lines represent results obtained for $q_1=0.1$, while the dash lines are the results for $q_1=0.3$. Other parameters are set as in Tables 1, 2

**Fig. 5:**
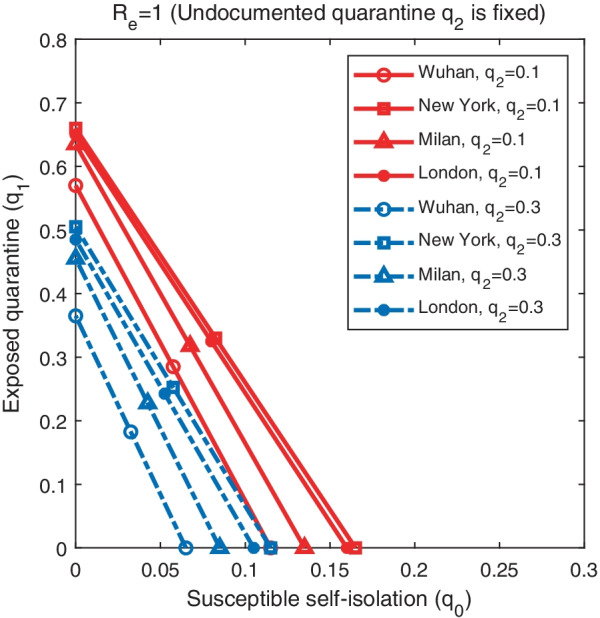
The combination of $q_0$ and $q_1$ that lead to $R_e=1$ in Wuhan, New York, Milan, and London. The solid lines represent results obtained for $q_2=0.1$, while the dash lines are the results for $q_2=0.3$. Other parameters are set as in Tables 1, 2

We performed similar analyses by fixing $q_1$ (Fig. 4) and $q_2$ (Fig. 5). In order to reduce $R_e$ below 1, undocumented infections or exposed individuals need to be quarantined with a much faster quarantine rate than the self-isolation of susceptible population. This pattern consistently holds across all four cities. The required self-isolation rate of susceptible population $q_0$ decreases with increased quarantine rate of undocumented infection $q_2$ and exposed individual $q_1$. In order to minimize the population size under self-isolation and reduce the disturbance on society, the best control strategy should be to isolate confirmed cases and individuals who are exposed to infections (possible undocumented infections) as soon as possible so that the required self-isolation rate of susceptible population could be lower.

We further explore the impact of self-isolation rate $q_0$ and quarantine rates $q_1$ and $q_2$ on several characteristics of the outbreak, including the outbreak duration, attack rate, and peak timing. Here the outbreak duration is defined as the number of days it takes for daily cases to drop below 5 after the lockdown measures are enforced; the attack rate is the percentage of population infected with SARS-CoV-2 by the time daily cases fall below 5; and peak timing is the number of days between lockdown and the day with the highest reported daily case. We ran model simulations using different combinations of self-isolation rate $q_0$ and quarantine rates $q_1$ and $q_2$ starting from lockdown with other parameters set as in Tables 1, 2, until the daily case number falls to 5.

**Fig. 6:**
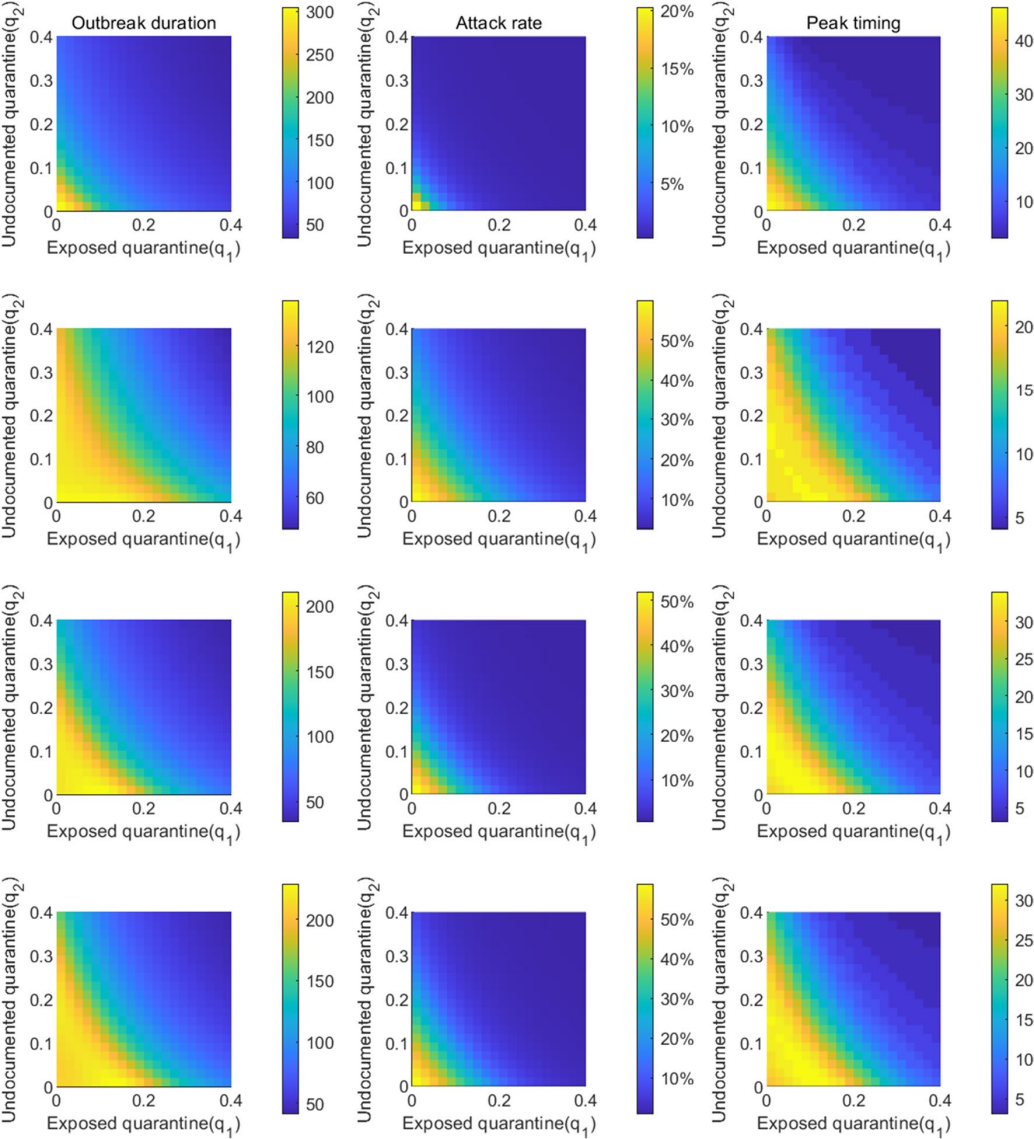
Impact of quarantine rates $q_1$ and $q_2$ on the outbreak duration, attack rate, and peak timing. The self-isolation rate $q_0$ is set as 0.03. The first column shows the duration of the outbreak, i.e., the number of days it takes for daily cases to drop below 5 after the lockdown measures are enforced; the second column shows the attack rate by the time when daily cases fall below 5; the third column shows peak timing of daily cases after lockdown, defined as the number of days between lockdown and the day with the highest reported daily case. Each row corresponds to one city

**Fig. 7:**
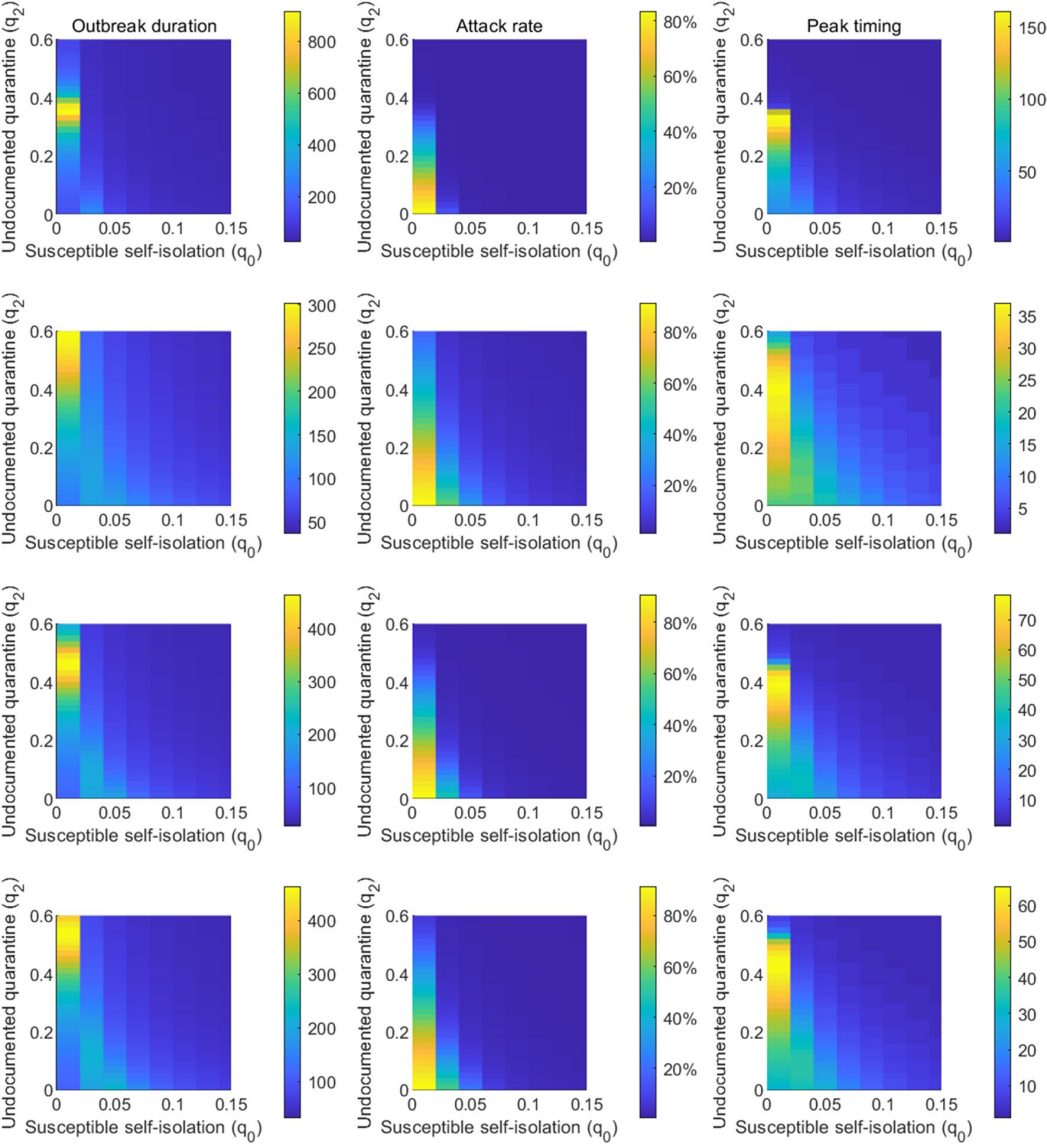
Impact of self-isolation rate $q_0$ and quarantine rate $q_2$ on the outbreak duration, attack rate, and peak timing. The quarantine rate $q_1$ is set as 0.1. The first column shows the duration of the outbreak, i.e., the number of days it takes for daily cases to drop below 5 after the lockdown measures are enforced; the second column shows the attack rate by the time when daily cases fall below 5; the third column shows peak timing of daily cases after lockdown, defined as the number of days between lockdown and the day with the highest reported daily case. Each row corresponds to one city

**Fig. 8:**
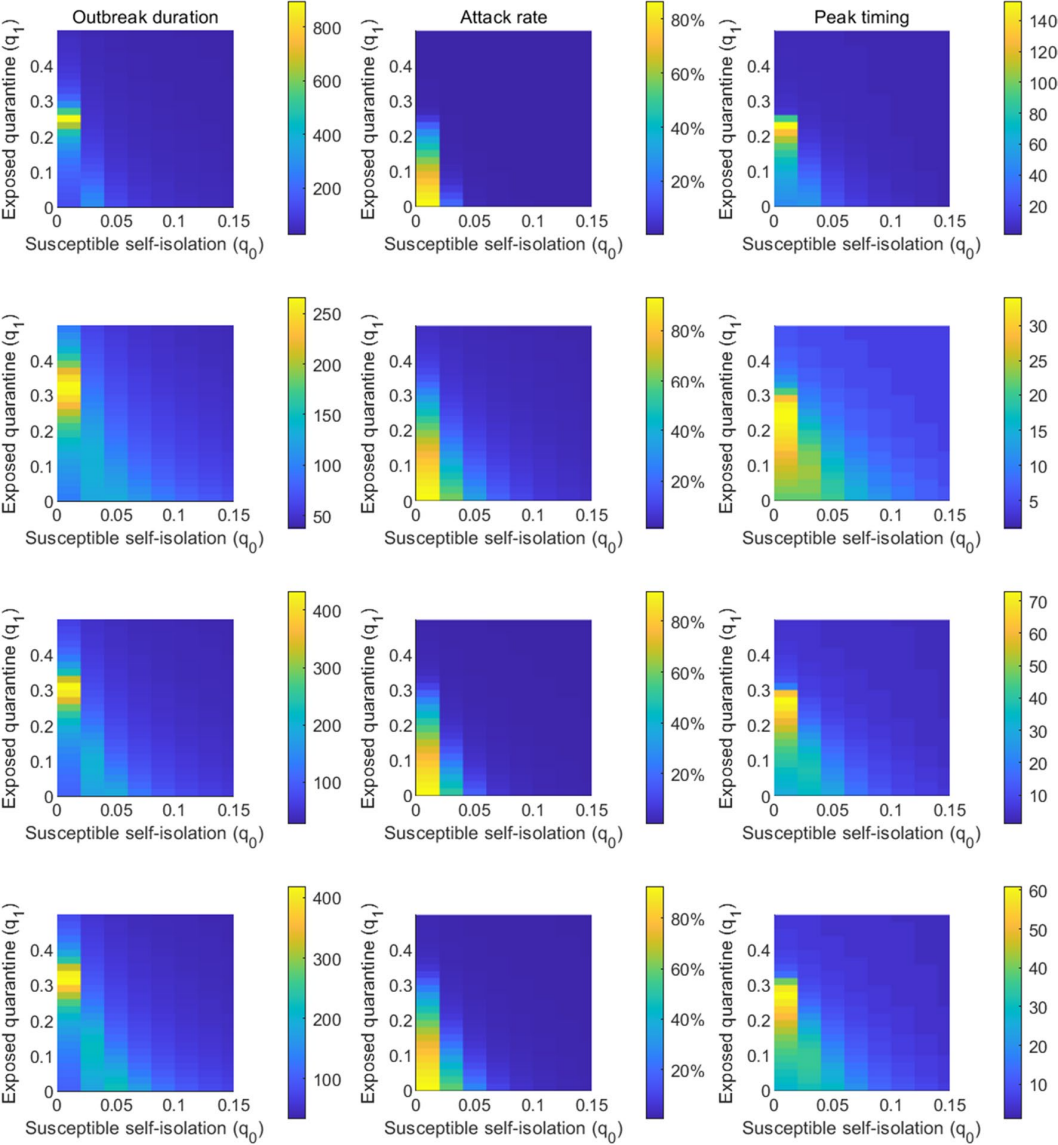
Impact of self-isolation rate $q_0$ and quarantine rate $q_1$ on the outbreak duration, attack rate, and peak timing. The quarantine rate $q_2$ is set as 0.1. The first column shows the duration of the outbreak, i.e., the number of days it takes for daily cases to drop below 5 after the lockdown measures are enforced; the second column shows the attack rate by the time when daily cases fall below 5; the third column shows peak timing of daily cases after lockdown, defined as the number of days between lockdown and the day with the highest reported daily case. Each row corresponds to one city

We first fixed the self-isolation rate $q_0$ at 0.03 and varied quarantine rates $q_1$ and $q_2$. Simulation results are shown in Fig. 6. The outbreak duration is maximized for the combinations of $q_1$ and $q_2$ that lead to $R_e=1$. For $R_e>1$, the outbreak depletes susceptible population and stops due to herd immunity; for $R_e<1$, the outbreak dies out as the low secondary infection rate cannot support self-sustained transmission, leaving the majority of population susceptible. At the critical state $R_e=1$, the outbreak would linger for a long period until herd immunity stops disease spread. Peak timing also follows the same pattern, as shown in the right column of Fig. 6. The outbreak duration and peak timing is shorter in cities with higher $R_e$ before lockdown. Attack rate increases with lower rates $q_1$ and $q_2$. Without control, over 20% population in Wuhan would be infected, while 50% population in the other three cities. We repeated the same analysis for fixed $q_1=0.1$ (Fig. 7) and $q_2=0.1$ (Fig. 8).

### Estimating the averted cases due to quarantine measures

**Fig. 9:**
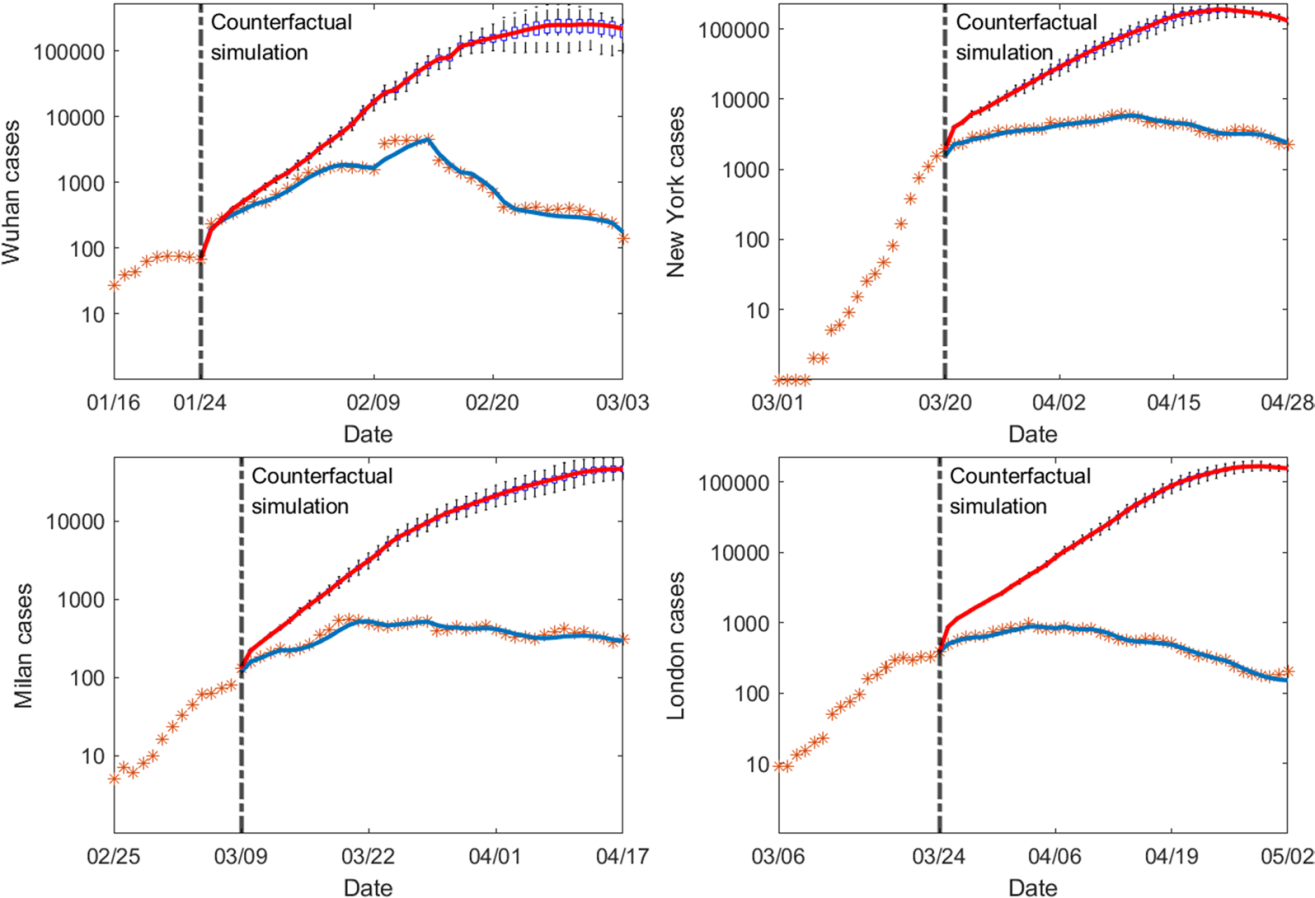
Counterfactual simulations of outbreaks in Wuhan, New York, Milan and London assuming no stay-at-home and quarantine measures. Simulations were performed using posterior model parameters estimated each day in the four cities within 40 days after lockdown, with the self-isolation rate $q_0=0$ and quarantine rates $q_1=q_2=0$. The vertical dash lines show the starting dates of counterfactual simulations. The orange stars are the observed daily case numbers. The blue curve is the mean posterior fitting using the EAKF. Blue boxes show the median and interquartile of counterfactual simulations, and whiskers show 95% CI. The solid red lines are the median of counterfactual simulations. Counterfactual simulations were performed for 100 realizations using independently estimated model parameters

We ran counterfactual simulations starting from the date of lockdown in each city assuming no stay-at-home and quarantine measures were implemented ($q_0=q_1=q_2=0$). We compare the counterfactual simulation outcomes with observed cases numbers in Fig. 9. Without quarantine measures, the outbreak would get out of control and result in massive disease spread. In total, we estimated that the quarantine measures have averted 3,589,622, 3,281,480, 629,046 and 2,452,750 confirmed cases in Wuhan, New York, Milan, and London during the 40-day period after lockdown. In other words, the cumulative case number would be 71, 21, 41 and 99 times higher than the reported number during this period in Wuhan, New York, Milan, and London. These counterfactual simulations indicate that strict stay-at-home and quarantine measures are essential to control the spread of COVID-19 during the early phase of the pandemic.

In addition, we ran counterfactual simulations in which self-isolation of the susceptible population is not enacted, that is, $q_0=0$, and other parameters remain unchanged as estimated. The results showed that, compared with the counterfactual scenario that quarantine is in place but stay-at-home is not effected, 570,696 confirmed cases were averted by self-isolation in Wuhan, 283,020 in New York, 21,255 in Milan, and 81,737 in London. Compared with the results in Fig. 9, the averted cases are much lower. This indicates that stay-at-home orders need to work in synergy with quarantine to effectively limit COVID-19 spread.

## Conclusions

In this study, we developed an SEIR-type disease transmission model to evaluate the impact of stay-at-home and quarantine measures on COVID-19 spread in four cities that experienced early large-scale outbreaks - Wuhan, New York, Milan and London. Using the transmission model in conjunction with data assimilation techniques, we estimated key epidemiological parameters in each city before lockdown. We examined the impact of stay-at-home and quarantine on COVID-19 spread after lockdown by adjusting self-isolation and quarantine rates in counterfactual simulations. We found that quarantine of susceptible population is necessary to contain the outbreak. Self-isolation of susceptible population induced by stay-at-home orders is more effective in reducing effective reproductive numbers $R_e$. Variation in self-isolation and quarantine rates can also considerably affect the duration of outbreaks, attack rates and peak timing. We generate counterfactual simulations to estimate effectiveness of stay-at-home and quarantine measures. Without these two measures, the cumulative confirmed cases could be much higher than reported numbers within 40 days after lockdown in Wuhan, New York, Milan, and London.

There are several limitations in the study. First, we neglected the effect of quarantine of SARS-CoV-2 contacts on the susceptible population. In reality, the number of exposed susceptible individuals may increase with the number of infectious individuals. However, as only a small fraction of susceptible individuals have close contacts with infectious persons during the early pandemic, we believe this model simplification does not significantly affect our results. Second, we did not explicitly consider contact tracing efforts implemented after lockdown. The contact tracing capacity was limited at the beginning of the pandemic, and the effect of contact tracing can be implicitly represented by elevated ascertainment rate. Thirdly, we assume model parameters in counterfactual simulations such as the transmission rate and ascertainment rate remain the same as estimated using real-world data. However, human behavior may change in response to large-scale local outbreaks. Our counterfactual results are therefore conditioned on the idealized assumption that population behavior does not change except self-isolation and quarantine rates. Lastly, human behaviors and cultures vary in different counties and could impact the compliance with control measures. In this study, we focused on four metropolitan areas in developed settings. Results in developing countries may be different given potential differing behaviors and cultures.

## Supplementary Information

**Additional file 1.** Code to perform the analysis in the paper.

## Data Availability

The data of Wuhan is collected from the daily news published on the official website of the Hubei Provincial Health Commission: http://wjw.hubei.gov.cn. The New York City data is published by THE CITY at https://github.com/thecityny/covid-19-nyc-data, and their official website is https://thecity.nyc. The data for Milan is available at https://github.com/RamiKrispin/covid19Italy by Rami Krispin. The data for London is collected from https://coronavirus.data.gov.uk. All data are publicly available. The code is uploaded as the Additional file 1 and can be freely downloaded.
